# Euglycemic Diabetic Ketoacidosis in a Patient with Cocaine Intoxication

**DOI:** 10.1155/2016/4275651

**Published:** 2016-08-08

**Authors:** Asma Abu-Abed Abdin, Muhammad Hamza, Muhammad S. Khan, Awab Ahmed

**Affiliations:** ^1^Jordan University for Science and Technology, P.O. Box 3030, Irbid 22110, Jordan; ^2^Department of Medicine, Dow Medical College, Baba-E-Urdu Road, Karachi 74200, Pakistan; ^3^Section of Pulmonary & Critical Care Medicine, University of Oklahoma Health Sciences Center, 920 Stanton L Young Boulevard, Oklahoma City, OK 73104, USA

## Abstract

Diabetic ketoacidosis (DKA) is characterized by elevated anion gap metabolic acidosis, hyperglycemia, and elevated ketones in urine and blood. Hyperglycemia is a key component of DKA; however, a subset of DKA patients can present with near-normal blood glucose, an entity described as “euglycemic DKA.” This rare phenomenon is thought to be due to starvation and food restriction in insulin dependent diabetic patients. Cocaine abuse is considered a trigger for development of DKA. Cocaine also has anorexic effects. We describe an interesting case of euglycemic DKA in a middle-aged diabetic female presenting with elevated anion gap metabolic acidosis, with near-normal blood glucose, in the settings of noncompliance to insulin and cocaine abuse. We have postulated that cocaine abuse was implicated in the pathophysiology of euglycemic DKA in this case. This case highlights complex physiological interplay between type-1 diabetes, noncompliance to insulin, and cocaine abuse leading to DKA, with starvation physiology causing development of euglycemic DKA.

## 1. Introduction

The hallmark of diabetic ketoacidosis (DKA) is hyperglycemia, metabolic acidosis, and increased ketones [1]. The absence of hyperglycemia, defined as serum glucose concentration greater than 250 mg/dL [1], makes the diagnosis of DKA less obvious in a patient with elevated serum anion gap metabolic acidosis and positive serum and urine ketones. However, euglycemic DKA has been well described in the literature mainly in type-1 diabetic patients, starvation, gestational diabetes, and more recently the settings SGLT inhibitor use. We describe a case of middle-aged women, who presented with severe metabolic acidosis, ketonemia, and near-normal blood glucose in the settings of noncompliance to insulin and cocaine use leading to poor oral intake. This case report also reviews possible physiologic interplay between lack of insulin and cocaine use leading to DKA.

## 2. Case

**Figure 1:**
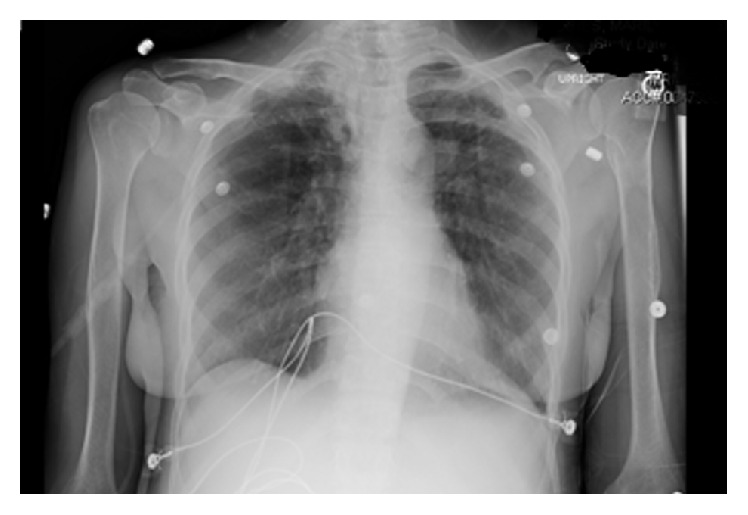
Chest X-ray on admission.

A fifty-seven-year-old female presented to emergency room with altered mental status, nausea, vomiting, and abdominal pain. The patient was in usual state of health 2 days before presentation, when she developed intermittent dull epigastric pain. The patient was able to tolerate the pain initially, but on day of presentation the pain became severe and persistent and she started having nonbilious vomiting. She also became somnolent and tachypneic and therefore the decision was made to bring her to the emergency room. Her past medical history was significant for diabetes mellitus and she was on insulin monotherapy. The family reported that for the last few weeks the patient was noncompliant with using insulin. On initial evaluation her vital signs were remarkable for temperature of 37 degree Celsius, pulse of 120/minutes, blood pressure (BP) of 134/78 mmHg, respiratory rate of 24 breath/minute, and oxygen saturation of 95% on 2-liter nasal cannula. Pertinent examination findings included obtunded patient who did not wake up to deep sternal rub, dry oral mucosa, and a weak gag reflex. Her pulmonary and cardiovascular examination was unremarkable. Admission labs indicated severe uncompensated metabolic acidosis with arterial blood gas showing pH of 7.02, PaCO_2_ of 14, PaO_2_ of 134, calculated bicarbonate of 4, and oxygen saturation of 97%. Serum chemistries indicated sodium 137 mEq/L (136–145 mEq/L), potassium 4.4 mEq/L (3.5–5.1 mEq/L), chloride 82 mEq/L (97–109 mEq/L), bicarbonate < 10 mEq/L (23–32 mEq/L), blood urea nitrogen (BUN) 32 mg/dL (7–17 mg/dL), serum creatinine 2.84 mg/dL (0.7–1.1 mg/dL), serum glucose 172 mg/dL (66–111 mg/dL), measured serum anion gap 46 (4–14), and measured serum osmolality 324 mOsm/kg (280–300 mOsm/kg). Her urine analysis was positive for ketones. Urine and serum drug screen was positive for cocaine. Other labs including complete blood count, lactic acid, serum Tylenol levels, and liver function tests were within normal limits. Admission chest roentgenogram was unremarkable (Figure 1).

Given concern for severe uncompensated metabolic acidosis and decreased sensorium, the patient was intubated for airway protection. Initial differential diagnosis included toxic alcohol ingestion, euglycemic diabetic ketoacidosis, alcoholic ketoacidosis, and starvation ketoacidosis. The serum ethanol, methanol, ethylene glycol, and propylene glycol were undetectable on admission labs and serum beta-hydroxybutyrate (BHB) levels were significantly elevated 15.95 mmol/L (0.02–0.27 mmol/L) confirming the diagnosis of euglycemic diabetic ketoacidosis. As the patient's blood glucose was 172 mg/dL on admission, we initially administered 1 liter bolus of intravenous (IV) 5% Dextrose and 0.45% normal saline (D5-1/2 NS), followed by a maintenance rate of 250 cc/hour, supplemented with 20 mEq of potassium chloride IV per liter of D5-1/2 NS. Insulin drip was initiated at a rate of 0.1 units/kg/hour. Finger stick blood glucose was checked hourly to maintain blood glucose between 150 and 200 mg/dL. Basic metabolic panel and BHB levels were checked at four-hour interval. After initial 4 liters of volume resuscitation, the patient's anion gap started to close and in 18 hours period her gap closed completely as shown in Table 1.

The patient was subsequently transitioned to subcutaneous insulin. Her mentation improved and she was extubated. After extubation patient confirmed that she was on a cocaine binge for 2 days and missed her insulin doses during that time. The patient was transferred out of medical intensive care unit and was discharged from hospital subsequently.

## 3. Discussion

Diabetic ketoacidosis (DKA) is defined by the American Diabetes Association as having a combination of hyperglycemia (glucose > 250 mg/dL), acidosis (arterial pH < 7.3 and bicarbonate < 15 mEq/L), and ketosis (moderate ketonuria or ketonemia) [1]. Normally, human bodies have balance between insulin levels and their counter regulatory hormones which include glucagon, epinephrine, cortisol, and growth hormone. Any factor that causes decrease in insulin levels or increase in its counter regulatory hormones or both will lead to development of DKA [1].

Rare cases of DKA have been found to occur in the absence of true hyperglycemia; such cases are called euglycemic DKA. This phenomenon was first reported by Munro et al., when 37 out of 211 studied DKA cases were found to be normoglycemic (defined as a serum glucose level of 300 mg/dL (16.7 mmol/L) or lower and a plasma bicarbonate level of 10 mmol/L or lower at presentation) [2]. Thereafter, these criteria have been adjusted to consider serum glucose level of 200 mg/dL as a threshold for the diagnosis of euglycemic DKA. The underlying mechanism of this rare entity is either decreased hepatic production of glucose during fasting states or enhanced urinary excretion of glucose induced by increase in counter regulatory hormones, with the former being the more important; that is, when a diabetic patient is exposed to any triggering factor for DKA and is fasting for any cause while continuing his insulin treatment regularly, the liver will be in a state of glycogen depletion, which in turn leads to decreased hepatic glucose production and increased lipolysis and fatty acid production, and this finally will lead to decreased glucose production while ketone bodies formation is continuous, ending in a state of euglycemic DKA [3]. Some of the causes that have been reported to induce euglycemic DKA include low caloric intake and starvation or prolonged vomiting, pregnancy, pancreatitis, insulin pump use, and SGLT2 inhibitors [4–9].  Table 2 gives major differentiating points between hyperglycemia DKA and euglycemic DKA.

Euglycemic DKA can be distinguished from other forms of ketoacidosis including starvation ketoacidosis, and alcoholic ketoacidosis, by clinical history and serum bicarbonate levels. The serum bicarbonate concentration in starvation ketoacidosis is usually more than 18 mEq/L [10]. The absence of other causes of elevated anion gap metabolic acidosis such as lactic acidosis, elevated toxic serum alcohols (methanol, ethylene glycol, etc.), drug toxicity, paraldehyde ingestion, and renal failure can distinguish DKA.

In the current case, the patient presented with severe uncompensated metabolic acidosis, ketonuria, and ketonemia in the absence of true hyperglycemia. After excluding other possibilities of wide anion gap metabolic acidosis including ethanol, methanol, or ethylene glycol ingestion, the diagnosis of euglycemic DKA was made and it was treated accordingly by IV 5% Dextrose, 0.45% normal saline, and insulin drip. The patient was screened positive for cocaine in serum and urine drug screen, and thereafter she admitted that she had been on binge cocaine intake for two days before presentation. Interestingly, cocaine has been reported to be a trigger for DKA in many diabetic patients. This is attributed to cocaine's stimulatory effect on cortisol, epinephrine, and norepinephrine release from the adrenal gland [11, 12]. This increase in counter regulatory hormones is the underlying cause for having DKA with hyperglycemia in such patients.

In this patient, two counter regulatory factors played a role culminating in development of euglycemic DKA. We hypothesize that, with history of noncompliance to insulin and while being on cocaine binge, the patient developed hyperglycemic DKA for two reasons: firstly, from lack of exogenous insulin and secondly from hyperglycemic effect of cocaine via adrenocortical hormones as described above. Conversely, as cocaine has been shown to cause anorexic effect via suppression of feeding centers in central nervous system [13, 14] and while being on cocaine binge, the patient developed poor oral intake. This starvation effect led to drop in her blood sugar to normal range. Interestingly, the anorexic, thus, hypoglycemic effects of cocaine were more pronounced in our patient, hence overshadowing the initial hyperglycemia resulting in euglycemic DKA.

In conclusion, euglycemic DKA should be included in differential diagnosis in patients presenting with elevated anion gap metabolic acidosis and near-normal blood glucose, especially in the setting of cocaine abuse. Volume resuscitation and insulin drip initiation remain the cornerstone of treatment and early diagnosis through high index of suspicion is paramount in order to start the right treatment.

## Figures and Tables

**Table 1:** Basic metabolic panel, beta-hydroxybutyrate, and serum pH from time of admission to closure of anion gap.

	Admission	Six hours apart	Four hours apart	Four hours apart	Four hours apart	Four hours apart
Sodium	137 mEq/L	139 mEq/L	139 mEq/L	138 mEq/L	138 mEq/L	137 mEq/L
Potassium	4.4 mEq/L	4.8 mEq/L	4.3 mEq/L	4.1 mEq/L	3.9 mEq/L	4.2 mEq/L
Chloride	82 mEq/L	102 mEq/L	103 mEq/L	105 mEq/L	107 mEq/L	107 mEq/L
Bicarbonate	<10 mEq/L	<10 mEq/L	10 mEq/L	15 mEq/L	21 mEq/L	22 mEq/L
Blood urea nitrogen	32 mg/dL	25 mg/dL	22 mg/dL	19 mg/dL	15 mg/dL	14 mg/dL
Creatinine	2.84 mg/dL	1.82 mg/dL	1.69 mg/dL	1.66 mg/dL	1.20 mg/dL	1.06 mg/dL
Glucose	172 mg/dL	251 mg/dL	180 mg/dL	198 mg/dL	166 mg/dL	95 mg/dL
Anion gap	46	28	26	18	10	8
B-Hydroxybutyrate	—	15.95 mmol/L	—	4.51 mmol/L	—	0.05 mmol/L
pH	7.02	7.13	7.26	7.34	7.36	7.38

**Table 2:** Major differences between hyperglycemic DKA and euglycemic DKA.

Hyperglycemic DKA	Euglycemic DKA
Blood glucose > 250 mg/dL, arterial pH < 7.3, and serum bicarbonate < 18 mEq/L	Blood glucose < 200 mg/dL, arterial pH < 7.3, and serum bicarbonate < 18 mEq/L
Insulin deficiency and increased counter regulatory hormones causing hyperglycemia	Insulin deficiency and increased counter regulatory hormones causing hyperglycemia and starvation from any cause euglycemia
Triggered by infections, concurrent illnesses, and dehydration	As a result of decreased circulating glucose in starvation, cirrhosis, exogenous insulin use, SGLT-2 inhibitors, pancreatitis, depression, and so forth
